# Organ-On-A-Chip: A Survey of Technical Results and Problems

**DOI:** 10.3389/fbioe.2022.840674

**Published:** 2022-02-10

**Authors:** Alex Ede Danku, Eva-H Dulf, Cornelia Braicu, Ancuta Jurj, Ioana Berindan-Neagoe

**Affiliations:** ^1^ Department of Automation, Technical University of Cluj Napoca, Cluj-Napoca, Romania; ^2^ Research Center for Functional Genomics, Biomedicine and Translational Medicine, “Iuliu Hatieganu” University of Medicine and Pharmacy, Cluj-Napoca, Romania

**Keywords:** organs-on-a-chip, microfluidics, personalized medicine, technical details, nanotechnology

## Abstract

Organ-on-a-chip (OoC), also known as micro physiological systems or “tissue chips” have attracted substantial interest in recent years due to their numerous applications, especially in precision medicine, drug development and screening. Organ-on-a-chip devices can replicate key aspects of human physiology, providing insights into the studied organ function and disease pathophysiology. Moreover, these can accurately be used in drug discovery for personalized medicine. These devices present useful substitutes to traditional preclinical cell culture methods and can reduce the use of *in vivo* animal studies. In the last few years OoC design technology has seen dramatic advances, leading to a wide range of biomedical applications. These advances have also revealed not only new challenges but also new opportunities. There is a need for multidisciplinary knowledge from the biomedical and engineering fields to understand and realize OoCs. The present review provides a snapshot of this fast-evolving technology, discusses current applications and highlights advantages and disadvantages for biomedical approaches.

## 1 Introduction

**FIGURE 1:**
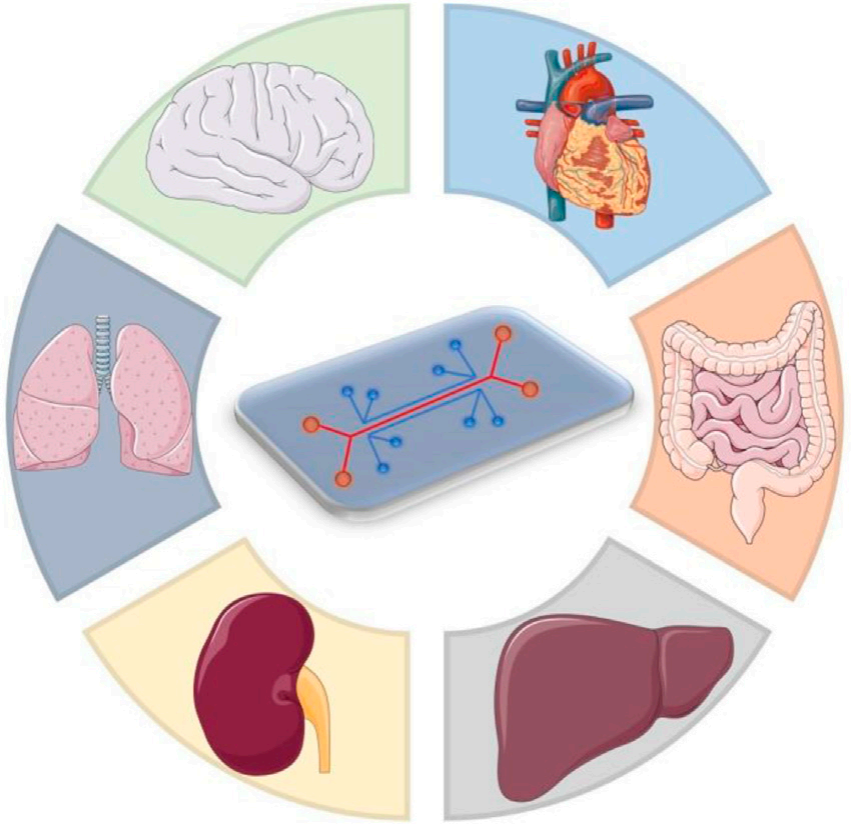
Multi-organ chip platform for disease modeling.

Organ-on-chip (OoC) is a concept with great interest all around the globe, due to the importance of their applications in biomedical field. The principle of the OoC is to emulate the behavior of different cells, organs and even multiple-organs, to mimic the complex physiological or pathological processes (Mauriac et al., 2017c; Sun et al., 2019; van den Berg et al., 2019). From an engineering point of view, OoC is a microfluidic cell culture system with controlled conditions (flow, velocity, etc.) that imitate the physico-chemical microenvironment of tissues in the human body (Figure 1). A difficult goal which needs to be achieved is the body-on-a-chip concept, which requires multiple OoC of different cell type or organs to be linked in order to create a system (Lee et al., 2018). OoC has several applications, but the most important is drug development and the effects that they have on different organs. Drugs are mostly tested on animals, which in some cases give inaccurate data or raise ethical concerns from organizations such as People for the Ethical Treatment of Animal (PETA), but it is considered an important step in tumor research (Mattei et al., 2021). This led to researchers searching for new ways to allow testing on human cells (Wikswo et al., 2013; Marx, 2016; Zheng et al., 2016; Kodzius et al., 2017; Jusoh et al., 2019). The concept of OoC is revolutionary, having a “mini-me” (personalized chip) on which different drugs can be tested on a system similar to that of the patient, making it possible to detect the correct drug without harming the patient, in same time permitting the selecting of optimal treatment that would be useful for the selected OoC design (Zhang and Radisic, 2017; Ogden et al., 2021). OoC uses at its base the control of microfluids, which are generally restricted geometrically to a small quantity of fluid in the range of nanoliters. The liquids in question are found inside small ducts of dimensions ranging from tens to hundreds of micrometers (Singh et al., 2017; Zhang et al., 2018). On the long term the use of this technology may become the means of successfully treating individual patients by using their own cells to develop personalized medicine approaches (Satoh et al., 2018). Cancer cell behavior analysis can be done by replicating the tumor cell behavior in these OoC. At the same time, they offer the possibility to experiment and determine the progress and the response for developing novel therapeutic strategies (Hachey and Hughes, 2018; Rodrigues et al., 2020). For each organ a different microchip is developed, since they need to replicate complex structural and cellular interactions in and between diverse cell types and organs (Huh et al., 2012; Wikswo et al., 2013; Mauriac et al., 2017c; Zhang and Radisic, 2017). Also, OoC can be used as well to mimic healthy tissue permitting the exploration of fundamental mechanisms in physiology and drug toxicity screening (Ribas et al., 2017; Raje and Raje, 2019). In recent years, microfluidics has reached the Lab-on-a-chip level, where drugs are tested for their effectiveness. In the pharmaceutical, field OoC is used to identify and detect different drug molecules both in quantity and quality. Some of the limitations of the pharmaceutical analysis have been removed giving access to more applications such as drug screening, drug quality control and precision medicine (Jia et al., 2022).

In the next part of the paper some of the OoC developed for different organs will be discussed. The advantages and disadvantages of OoC will be presented and the structure and elements of such a chip will be discussed. Going further we will discuss some of the microsystem types used in the OoC applications analyzing different organs, considering the 3D structure and cellular composition. Before the conclusion, the concept of personalized OoC and how it can be used will be addressed.

## 2 Strengths and Limitations of OoC

In the following part the advantages are discussed reasoning the use of the OoC technology, while later some of the limitations of the concept will be discussed.

The first major strong point of the organ-on-a-chip concept is the accelerated research it can generate (Tran et al., 2021). Since the cost of manufacturing the chips is rather cheap, it is possible to fabricate using in-house accessories without any specialized equipment (Abu-Dawas et al., 2021), many drugs and doses of drugs can be tested at the same time. This may prove helpful when a new drug is discovered, not needing test subjects and at the same time not meeting ethnic concerns. Another strong suit of the OoC concept is the close resemblance of the tissue microenvironment it replicates. When comparing the OoC with simple Petri recipient microsystems, the OoC comes out on top due to the 3D structure which is an important element of the test’s reliability. Additionally, the microfluidic chips are user friendly and, in some cases, can be portable are capable to assess many physiological questions. Due to their small size multiple microfluidic systems can be integrated on one chip, saving space and money at the same time.

The first disadvantage considered is the presence of the surface effect. Since the dimensions of the fluids are very small, the surface effects dominate the volume effect. This may reflect in poor quality of the analysis and some of the product of interest may be adsorbed. Since laminar flow is present at the intersection of multiple fluids the relevant fluids might not mix properly. Another limitation of these platforms is represented by the fact that, in some experiments, there is a need for special instruments in order to obtain reliable results (Mauriac et al., 2017c; Bein et al., 2018; Sontheimer-Phelps et al., 2019).

## 3 Materials Needed and Outside Devices

The organ-on-a-chip needs to be manufactured using a material which does not influence the cellular microenvironment components and maintain a stable fluid connection (Vassanelli, 2011; Huh et al., 2013). The most popular material used is known as polydimethylsiloxane (PDMS). This material is a polymetric and synthetic elastomer based on carbon and silicon. The manufacturing itself is made of mixing the liquid PDMS with an agent which helps with the solidification of PDMS. The mixture is then poured into a mold giving the form of the chip. After the mixture hardens the chips, body can be either glued to lass or to another chip. The PDMS became popular due to many of its properties, being transparent it helps the user since they can see how the OoC behaves. The material is cheap and is known to have a reduced cytotoxicity, making it easy to use in this application. Because PDMS doesn’t degrade it is sometimes replaced by other materials (Ahadian et al., 2018; Sosa-Hernández et al., 2018). However there have been researchers who tried to 3D print the chip, requiring only one step, and avoiding any troublesome events that may occur using multiple step methods that would require the solubilization of the initial scaffold (Lee and Cho, 2016).

Due to the different structure of each organ, different biomaterials are required in order to obtain good results. For example, collagen is widely used because of its advantages, but it requires some mechanical support without which the collagen remains intact for a short time. In some cases, external equipment is required in order to obtain the best results. Firstly, it is necessary to control the external flow of the micro and nanofluids. For this pressure generators and different types of pumps are used (Ho et al., 2015; Mauriac et al., 2017b). The best way to control flow is to use hydrostatic pressure. Pressure generators are usually simple devices, having incorporated a pressure source, for example a compressor, a pressure regulator and a manometer to measure the current pressure value. Even though the system is simple it does have major setbacks mostly contained in its limited response time (Perestrelo et al., 2015; Mauriac et al., 2018). There are, however, methods to bypass this problem, using a pressure multiplexer the pressure can be changed much faster. Another upgrade to pressure generators can be done by adding flux sensors which change the control signal from pressure to flow (Esch et al., 2016; Mauriac et al., 2017a; Wu et al., 2020). The viscosity and density of the cell culture changes after a certain number of days. When constructing the chip this property has to be remembered since studies show that the pressure and stress of the walls increased significantly in the analyzed models.

Pressure syringes are another system usually used for flow control. They are usually used in perfusions, but scientists have also adopted them in microfluidic research. They have the advantage of being able to control the flow without being affected by perturbations caused by fluid resistance. Same as the pressure generator, the settling time is high because of the small values the flow pulses use (Mauriac et al., 2017b; Mauriac et al., 2018).

Commonly, there are two types of pumps used for flow control, the first one being simple pumps used for liquids which have the disadvantage of having a nonlinear model. However, there exists a linear equation, used as an alternative in order to model the system easier. The systems usually require a good sensor in order to detect small flow fluctuations. The other type of pumps are the electro-osmotic ones, which do not have flow fluctuation problems and are resistant to high counter pressure, but have the disadvantage of requiring low conductivity liquids in order to work properly (Mauriac et al., 2017b; Wu et al., 2020).

## 4 Analyzed Organs

Organs are made up of different types of cells corresponding to their role in the human body. Since different organs have different roles the structure of their cells is changes depending on cell types. Thus, the OoC must be constructed to best suit the microenvironment the cells experimented on (Mertz et al., 2018).

### 4.1 Liver

The liver is one of the most important organs in the human body, mostly for its several functions to maintain normal physiological activities. In order to cope with the damages which may be caused to it through chemical or physical means, it has great regenerative capacity. In some cases, the injuries may be too severe, mostly caused by adverse reactions from different drugs or diseases. Before the OoC technology there were not many great *in vitro* models to be used. Most of the drugs were tested *in vivo* on animal subjects. The subjects are exposed to the adverse reactions of the drugs and, in some cases, being fatal to them. The OoC technology detects if the different drugs harm liver cells. The methodology is to seed the chip with liver cells and apply the drug in different channels. The chip is observed and if the cells die the drug is deemed to harmful to be used *in vivo*. Because of this, it can be considered that this system can be useful for hepatotoxicity studies (Wikswo et al., 2013).

There are several liver-on-chip methods used: Liver Sinusoid which replicates the lacuna between adjacent liver plates; Liver Lobule, considered the smallest functional unit of the liver; Zonation in the lobule is an optimized segregation of the liver functions in spatial and temporarily defined zones (Wikswo et al., 2013).

The engineering behind the liver-on-chips requires to integrate scaffolding materials for achieving 3D cultures, for cell growth and to maintain the interactions between the cells. Those components may be natural or synthetic, but are practiced in many applications. Such applications may vary from drug testing to behavior analysis. On a microfluidic chip it was observed that coating the chip with collagen supports the hepatocyte growth and adhesion. For a layer-by-layer deposition the cells were coated with nanofilms of fibronectin and gelatin which resulted in the reconstruction of liver tissue with high cellular function. Others found that by seeding hepatocytes, endothelial cells and stellate cells on fiber membranes the tissues formed secreted albumin and urea for several days (Kimura et al., 2018).

Most liver-on-a-chip models that study nanotoxicological effects are 2D systems. One of the more predominant roles of the liver is to filter the blood and remove the toxins from it. Due to the nature of this task the toxins can accumulate in the liver causing changes to its function and cellular morphology. One of the improvements to the liver-on-a-chip concept include the use of perfusions, the effect being the prolonged lifespan of the cells and improved drug metabolism. An innovative idea is the use of multi-species liver-on-a-chip system which uses the liver cells from multiple animals, this is done to prove that each species can react differently to certain drugs. In the example given, the drug killed the human liver cells, but the rat cells remained alive (Lu and Radisic, 2021).

### 4.2 Breast Tissue and the Tumor-On-A-Chip Concept

Understanding breast cancer anatomy is useful to better study breast cancer, a frequent malignancy among women. Its danger comes from its invasive properties and the difficulty in curing it. Different 2D models have been used throughout the years with little success, due to the models not being able to replicate in an accurate way the manner in which the tumor behaves. As in many other fields different *in vivo* experiments were studied on animals with the downside of these models being the lack of reproducibility of the obtained results (Bano, 2017).

The concept of tumor-on-chip (ToC) is a microfluidic 3D system designed to mimic the tumor behavior, biological activities, mechanical properties and different responses of the tumor cells. In the case of breast cancer these ToC devices are used for drug screening by studying different chemotherapeutic drugs and its effects on the model, or for personalized medicine when using primary cells (Chen et al., 2018). Knowing the factors that can induce resistance to tumor cells can lead to the discovery of new therapeutic strategies for treating this disease. With this knowledge, the risks of the treatments can be reduced and personalized drugs can be the next step of curing malignancies. For example, these models can be used to study the interaction of the malignant clone with adipocytes considering that this type of cells are prone for tumorigenesis and can promote drug response (Gao et al., 2020).

### 4.3 Pancreas

Pancreatic cancer is one of the types of cancer having one of the worse prognostics, due to its high resistance to the drugs used on it and the difficulty of removing the tumor through surgery. The poor outcomes of this disease are believed to be caused by the cancer cells which have high invasiveness features, thus the cancer reaching advanced stages in a short period of time (F. De Miollis et al., 2019).

Researchers took the OoC approach by using clear, flexible plastic chips which contained microfluidic channels. In order to replicate the behavior of the disease they use pancreatic cancer cells harvested from mice in one channel and seed the neighboring channel with human endothelial cells. The chip is then observed in order to study the behavior of these cells. After the experiment is concluded the same procedure is executed, but instead of mouse pancreatic cancer cells, human cells are used. The same behavior is seen suggesting that across different species the behavior for the disease does not change. This discovery means that whatever treatment is used on test subjects, such as mice, would work on humans as well. OoC are used in the search for a cure as well, by adding experimental cures to the cells of the chip then observing their behavior. In some cases, the spread of the cancer cells is slowed indicating that that substance should be tested *in vivo*.

Diabetes is a common disease that has a compromising effect on the functioning of the pancreas. There are several ways of better understanding this disease, one of the methods is with the aid of the OoC. The researchers use OoC technology to study the behavior of insulin producing cells in order to better understand the phenomenon. It is expected that with help of OoC the efforts of studying and understanding the disease should be accelerated (Wu et al., 2018; Glieberman et al., 2019).

### 4.4 Lungs

Lungs do not possess great regenerative properties, as such any damage caused to them might be permanent. In addition, pulmonary cancer is one of the more common types of cancers that may occur to a human, smokers being especially affected by this disease. Also, many areas have a high degree of air pollution, such as china, causing heavy damage to the lungs. Under these conditions the research on lung cells is deemed important. The increasing popularity of OoC means that researchers already try to model the lungs on this technology. There are certain researchers who accomplished this task using microfluidic devices and harvesting lung cells on these devices. They simulate the behavior of the lungs by increasing and decreasing the pressure of the device, while the cells replicate the behavior of the lungs (Wang et al., 2021). The researchers observed that the cells would react as if they were in their normal environment in the body, the alveola contracted and expanded mimicking the breathing, while the porous membrane expanded and contracted according to the pressure given. Having this technology, different reactions can be analyzed, ranging from the behavior of tumor cells to the reaction of lung cells to smoking or other environmental toxic agents. Different drugs can be tested as well to determine their effects on some diseases and to observe their potential side effects (Kimura et al., 2018).

OoC can be used to study the effects of different environmental factors on human organs. Such a study is done by Rick and Milica who analyze the adverse effects of nanoparticles and their toxicity. The nanoparticles are the remains from plastic and other non-biodegradable materials with the addition of fossil fuels and nitric oxide. It is proven that these have a toxic effect by the experiments done. Lungs exposed to these toxins are more likely to develop pulmonary complications like pulmonary fibrosis, asthma or pulmonary edema. Some of the problems occur due to the rapid advancements in technology and people not understanding how toxic can some of the newly developed fuels can be, should be considered for application on environmental toxicology. Most experiments regarding OoC are focused on either toxicity or diseases around the alveoli, only a fraction of the entire respiratory system, however there are different airway-on-a-chip models and even innovative approaches which use 3D printing and constructing in addition to the OoC concept (Lu and Radisic, 2021).

### 4.5 Brain

The brain might be the most complex organ and the hardest to create an OoC after. The functionality of the brain is far too complex and differs from person to person, as such in most papers the models do not cover this aspect, but rather go over and analyze cell ratios use the transporting properties of the brain and analyze the neuro vascular unit and the blood brain barrier. What makes the cell ratios of neurons and glial cells is that the ratio changes depending on the brain section which is analyzed (Wikswo et al., 2013).

There have been successful *in vitro* cell cultures in open environment such as glass substrates or Petri dishes. The technique used in the culture of the BoC is called multistep lithography. It consists of separating the soma and axon. The technique allowed the study of the axon, its regeneration and treatment using different drugs on the axon. The models created are used to study neurodegenerative diseases and understanding the behavior and the effects it has on the brain cells (Bang et al., 2019).

Three categories of BoC development are developed: 3D high content systems used to mimic the brain tissue environment; interconnected multichip system which is used to simulate interactions between multiple cells and organs; high-throughput systems, screening of experimental conditions.

### 4.6 Blood

The blood is made of different components: red blood cells, white blood cells, platelets and plasma. The models created with the organ on chips use these components in order to replicate the behavior of the blood. Each of the different blood cell type is added to different channels, being separated, due to each component having a separate role, from being part of the immune system components and emphasis the interaction among the key components. The hardest challenge is to recreate the circulatory system (Mao et al., 2020), since most cells mentioned before can be split into even more specific types (Ramadan and Gijs, 2015). The chip itself is made of a PDMS prepolymer through the photolithography process. The channels were 500 um to 1 mm wide. The obtained part is then bonded to a glass slide. Polystyrene beads are used to pack the channels (Wikswo et al., 2013).

These are just some of the organs which are modeled using the OoC technology. The list can be expanded to see the interaction with some organs, like kidneys and intestines, but the purpose of the paper is to present a wide variety of applications and to observe the development of the technology. The next challenge of the researchers is to realize the human body on a chip device. The previously mentioned devices take all the different organs on chip and model the functioning of the whole body. This is a challenge because bonding all the devices is a task of its own and obtaining the desired behavior from all the organs is not guaranteed.

Reports exist of the body-on-a-chip being realized by research groups. Multiple organ chambers are developed having different organ cells on the device which have to perform their physiological role (Table 1). The device is used on drug testing, observing the biological effects of the drugs on the whole complex system. The research team also obtained promising results for different anticancer drugs and successful kidney excretion functions. Even though this technology has been researched and there are successful results, the models created are still far from mimicking the whole human physiology to a greater extent. Currently there is a mathematical modeling approach being combined with the body-on-a-chip technology to recreate the human body behavior (REF).

**TABLE 1:** Summary for organs that have been recreated using organ-on-a-chip models.

Organ	Main characteristic	References
Liver	Maintains physiologic properties;	Wikswo et al. (2013), Kimura et al. (2018), Lu and Radisic, (2021)
	Great regenerative properties;	

Breast Tissue	Mainly used in cancer study and treatment;	Bano, (2017), Chen et al. (2018), Gao et al. (2020)
	Difficult to treat;	
Pancreas	Mainly used in cancer and diabetes study;	Wu et al. (2018), De Miollis et al. (2019), Glieberman et al. (2019)
	Hard to treat due to small number of vessels;	
Lungs	Bad regenerative properties;	Kimura et al. (2018), Lu and Radisic, (2021), Wang et al. (2021)
	Successful replication of functionality by modifying the pressure;	
Brain	Complex due to functionality;	Vassanelli, (2011), Wikswo et al. (2013), Bang et al. (2019)
	Differs from person to person;	
	Can be recreated by separating the soma and axons;	
Blood	Multiple cell types;	Wikswo et al. (2013), Ramadan and Gijs, (2015), Mao et al. (2020), Mandrycky et al. (2021)
	OoC uses all cell types;	
	Need for small chambers;	

In their study Christian et al. break down important aspects of the blood-on-a-chip concept. Since the blood vessels are organized in a hierarchical network it has different diameters as well as different roles. Some of the vessels are used to carry the oxygenated blood and are named arteries while the ones that carry the deoxygenated blood are the veins. The use of flow systems is required with this type of OoC, since laminar and disturbed flow has an effect on cytoskeletal model. These systems are also used to study blood clots. Over the years different microfluidic approaches have been found. For example, PDMS based structures went from modelling breathing and vascular models to anaerob intestine-on-a-chip and laying the foundation for the body-on-a-chip concept (Mandrycky et al., 2021).

## 5 Microsystem Types

### 5.1 Hydrogel Based Microsystems

**FIGURE 2:**
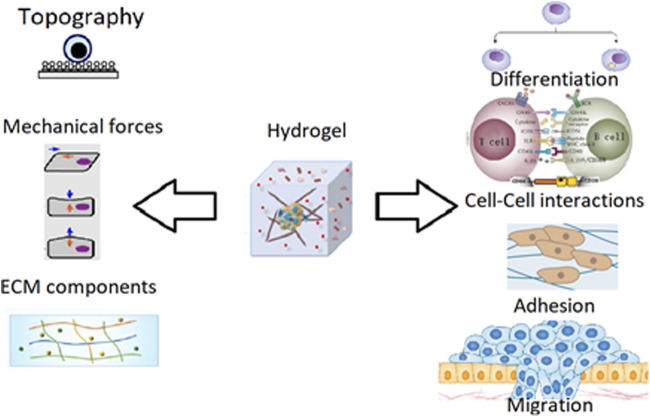
Hydrogel properties and behavior (Liu et al., 2019).

In their natural cells are surrounded by a network called Extracellular Matrix (ECM). The function and structure of the cells are given by the surrounding molecules. In cell culture this environment is missing, this results in different ways to mimic the environment. One of the more popular approaches uses the hydrogel (Figure 2) (Xie et al., 2020).

Hydrogels are made of different types of polymers which are swollen with water. Cells can be added inside the hydrogel by mixing the cell solution with the hydrogel before it gets solid. The cells are encapsulated in the gel which will be their ECM.

Hydrogels have different features which contribute to their popularity (Table 2). Such a feature is the possibility to place the cells in a 3D shape. The hydrogel can be customized to mimic the natural environment better. This can be done by adjusting the stiffness of the environment in order to match the cells natural environment. The main advantage however, is the absence of sophisticated materials or devices to enhance the cell growth. Furthermore, depending on the nature of the application the cells are going to be used for there are different types of hydrogels which can be used, from ECM based hydrogels to Natural biomimetic hydrogel. Hydrogels have been used to create different eye cells on chips. Studies show that the first attempt to recreate the cornea date back to 1993, while the first chip to model the cornea was done in 2009. In present days, however, more and more complex structures are made recreating each part of the eye, from the lacrimal glands to the retina (Manafi et al., 2021).

**TABLE 2:** Summary of hydrogel-based microenvironments.

Biomaterial	Advantage	Disadvantage	Type of OoC used in	References
Collagen	Biocompatible;	Need of chemical bonding for stability;	Cardiac;	Ahadian et al. (2018), Satoh et al. (2018), Sun et al. (2019), Arlk et al. (2021)
	Immunogenetic;	Need of mechanic support for the cells to remain intact;	Hepatic;	
	Enzymatic degradation;		Vascular;	
	Major component of ECM;		Kidney;	
	Cells can remodel the ECM gel;		Neurons;	
			Solid tumors;	
Fibrin	Biocompatible;	Weak mechanic properties;	Vascular tissue;	Wikswo et al. (2013), Ahadian et al. (2018), Sun et al. (2019)
	Noninflammable;		Lungs;	
	Biodegradable;		Skeletal muscles;	
	Gel modeling at room temperature;			
	Protein delivery system;			
Hyaluronic Acid	Biocompatible;	Weak mechanic properties;	Metastasis;	Rothbauer et al. (2018)
	Natural component of ECM;		Tissue barrier;	
	Structural component of tissues;			
	Regulable elasticity;			
Gelatin	Biocompatible;	Need of chemical bonding for stability;	Cardiac;	Ho et al. (2015)
	Biodegradable;		Vascular;	
	Similar composition as collagen;		Muscle;	
	Good elasticity;			
Synthetic biomaterials	Regulable mechanic properties;	Immune responses need to be evaluated;	Cardiac;	Ahadian et al. (2018), Sun et al. (2019)
	Less variable than natural biomaterials;	Can have cytotoxic effects;	Hepatic;	
	Chemically modifiable;			
	Degradable;			
	Can be modelled;			
Chitosan	Biocompatible;	Mechanical weakness;	Vascular;	Wikswo et al. (2013), Osório et al. (2021)
	Biodegradable;	Unstable;		
	Flexible;			
	Similar structure as glycosaminoglycans;			
Alginic acid	Biocompatible;	Uncontrolled degradation;	Cardiac;	Esch et al. (2016)
	Degradable;	Limited protein absorption;	Tumors;	
		Missing the binding cells;	Liver;	
			Marrow;	

Collagen is a main component of the ECM and is often used as biomaterial in OoC’s and are known for the key tissue-level functions like attachment and spreading (Arlk et al., 2021). It has many aiding properties. It is biocompatible and is known to have a controllable biodegradability which may be triggered by the use of enzymes. In medical applications it is mostly used to transport proteins and other growing catalysts, this property can be useful in OoC applications as well. Lastly, its cells can be remodeled and can be used to contract the ECM. There have been reports of using this biomaterial in the modeling of multiple organ and tissue types, from cardiac and hepatic to even different tumor cell types. On the other hand, it has some flaws, as it requires a mechanical support. In order to maintain stability a chemical coupling is required. All in all, the collagen is regarded as a very good biomaterial for OoC’s due to the wide variety of organs it can be used for (Ahadian et al., 2018; Satoh et al., 2018; Sun et al., 2019).

Fibrin is another biocompatible, nonflammable, biodegradable, biometric material. The gel can be made at room temperature. Similar to collagen, it has good protein transport properties and is considered a good bio-adhesive. It is used in the modeling of skeletal muscle tissue and vascularization. There are some researchers who could replicate the formation of fibrin cloths in lung models. The main downside of fibrin is its mechanical properties. Fibrin as a biometric material presents good properties, but has limited applicability compared to collagen (Wikswo et al., 2013; Ahadian et al., 2018; Sun et al., 2019).

For studying metastasis, hyaluronic acid can be considered as a biometric material, but similar to fibrin it has limited use and has bad mechanic properties. However, it is a natural component of the ECM and it is biocompatible for OoC applications. It is a structural component of tissues with a controllable elasticity (Rothbauer et al., 2018).

Gelatin is considered as a good replacement for collagen due to it having similar composition and shares some of its properties. It has been used to model cardiac, vascular and muscular tissues, but it is not used in such a wide area of applications as collagen. Such as its strengths, gelatin shares its disadvantages with collagen, since it requires chemical coupling for its stability (Ho et al., 2015).

Synthetic biomaterials, which can be classified as metals, ceramics, nonbiodegradable and biodegradable polymers, can be used to model cardiac and hepatic tissues and there are expectations of using them in the modelling of neurons. It is considered to have good mechanical and degradation properties. It is more reliable than other natural biomaterials. It uses polyesters which are known to degrade through hydrolysis. Can be reshaped easily and can be chemically modified to absorb bioactive cells. The main flaws of the synthetic biomaterials are the cytotoxic effect, degradation might cause. Also, the immune responses must be checked (Ahadian et al., 2018; Sun et al., 2019).

Chitosan is one of the most abundant polymers used in medical and bio chemical applications due to it being easily obtained from chitin. Its crystalline nature makes it possible to be processed with different methods in order to create gels, nanofibers and even sponges (Osório et al., 2021). It has good degradability, biocompatibility and low toxicity. It has been used in vascular, and even bone tissue modeling. The downsides of this biomaterial are the weak mechanical properties and it is considered unstable (Wikswo et al., 2013).

Alginic acid is known to be used as a medicine for hearth burns. It can be used as a biomaterial in OoC as a throwaway material. It is biocompatible and degradable, but its degradation cannot be controlled and it can absorb very limited amounts of proteins. It is used to model different tumors and cardiac, liver and marrow tissues (Esch et al., 2016).

### 5.2 Biometric Microfluidic Devices

**FIGURE 3:**
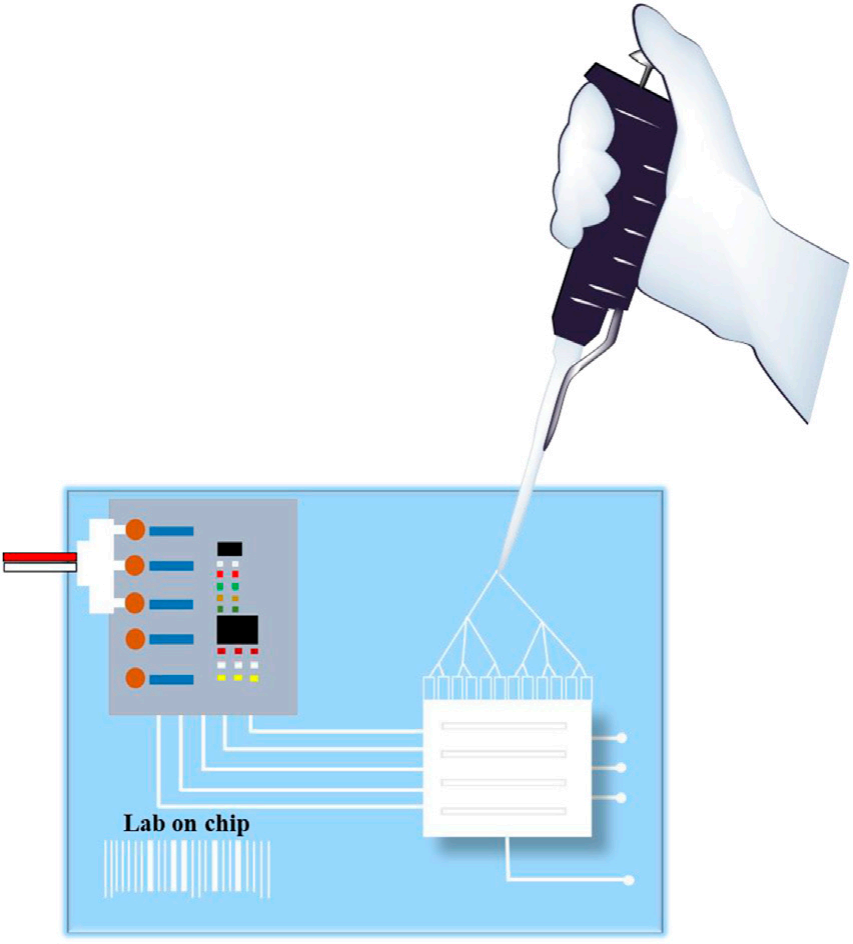
Organ-on-a-chip using microfluids.

Microfluidic devices are used in research centers, clinics and hospitals being powerful tools for monitoring, diagnostics and drug delivery. They were integrated in the OoC technology and revolutionized the fields of drug screening and toxicology studies (Figure 3) (Perestrelo et al., 2015).

The concept itself is associated with the control and manipulation of liquids at a small scale, as small as microliters. The advantages of microfluidics are vast: small sample volume, scalability, predictable fluid dynamics, high resolution and sensitivity, low cost, short analysis time and a large application pool. The applications vary from drug testing, to pollutant detection, going as far as repelling biowarfare adversities. In the OoC technology alone, microfluidics has a large number of useful applications: study of different organ and tissue activity, cell culture, drug delivery, wound healing, diagnostics (Bhise et al., 2014; Rothbauer et al., 2018).

Microfluidic devices are used for numerous different organs and systems of organs such as the respiratory system, excretory system, nervous system and many others. These systems consist of multiple organs, the devices containing different tissues and cells from each organ. For each system the role of the microfluidic devices may differ or they might have multiple roles (James Li and Zhou, 2013).

Even if the concept allows for breakthroughs, it has a lot of limitations. Most of them results from its complexity, but being on such a small scale it relates to the growth of cells too. Because the microfluidics is such a complex concept, the researcher needs to be skilled in order to perform the experiments. Until the concept is redesigned to be easier to understand this limitation will persist. Furthermore, the variability which is present from one batch to another is too big, this leading to a range of different results for the same parameters.

### 5.3 Devices Using Electrospinning

**FIGURE 4:**
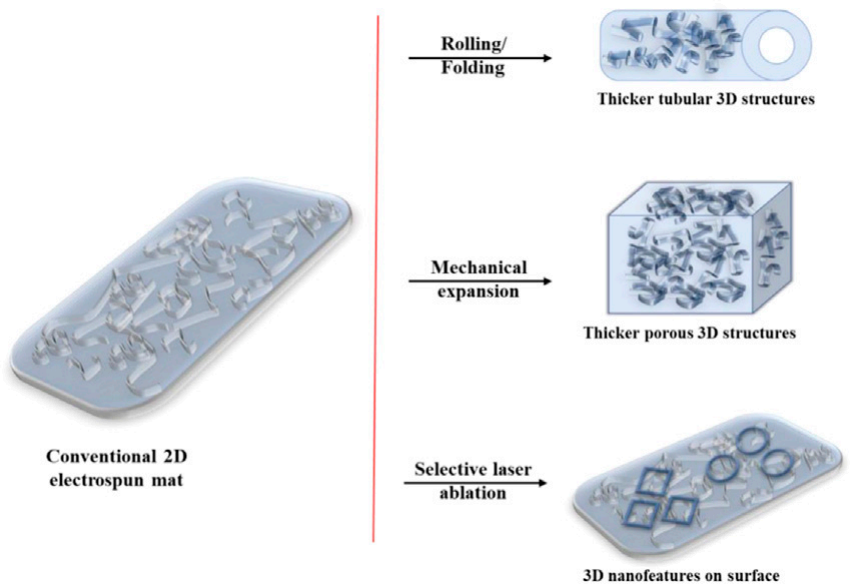
Electrospun mat possible applications.

Electrospun scaffolds have been increasing in popularity in recent years caused by their cost-effectiveness and the method being flexible (Figure 4). Their surface properties are different when compared to the base polymer sheets which are commonly used. The contact angle is increased due to the surface roughness. This problem can be solved by treating the polymers with nanofibers. The nanofibers are usually used to aid in the cell nutrient exchange, cell communication and efficient cellular responses. They are suited for this role since their size and morphology is similar to the human body’s extracellular matrix (Sampath Kumar and Yogeshwar Chakrapani, 2018).

Electrospinning requires a setup which in most cases consist of a high-voltage power supply, syringe pump, nozzle spinneret and a grounded drum collector. For this whole process to work properly there are several parameters which require some degree of precision. The drum collector for the setup used in the Koreans paper rotated at 30 rotations per minute, while the spinneret’s tip to collector distance was 100 mm. They used 10 kV electrical potential between the nozzle and the drum collector. Lastly the flow rate of the syringe should be maintained at 0.1 ml/h and the pressure, temperature and humidity need to be in a certain level. Depending on the application, the cells used and the equipment at disposal of the team these parameters may vary (Kiyoumarsioskouei et al., 2018; Kim et al., 2019).

### 5.4 Personalized Organs-On-A-Chip

Since the OoC research has known such a radical growth over the years, different more precise applications are being initiated. With the use of this concept an effort is being done in order to create personalized versions of the chip. This meaning that more specific and accurate diagnosis and drugs testing results can be obtained. The overall idea is to use as much information as possible when using the OoC, information specific to the individual the tissue sample is harvested. For example, in the experiments, the results will also be affected by genetic, physiological and biometric parameters specific to an individual. The obtained results give a clearer, more accurate model, or simulation, of how a disease, drug or specific external factors may react with the cells in the OoC, permitting the application of the concept of personalized medicine in clinical practice, selecting the optimal treatment scenarios for patient benefit (van den Berg et al., 2019).

Firstly, the precision treatment should be discussed. There are several factors which may make some drugs not eligible to use, ranging from pregnancy, age, diverse medical conditions and even being a smoker or not. There have been researchers that use OoC to compare the behavior of a healthy and of a smoker’s lungs (Kimura et al., 2018). Being able to give highly precise medication without risking the wellbeing of the patient may even save lives. There have been several reports and news of people receiving wrong medication because of the side effects the drugs they were given affected them differently due to the doctors not checking for a specific condition.

In order to test a drug on a control group, specific parameters are required and some conditional environments might be needed. If some patients need to be started on antibiotics for a bacterial infection, for each person several OoC will be used to test the effects of different antibiotics to check which one should be used with the least side effects. The patients might react differently to the medication they receive, with this method none of them risk to be mistreated (Raje and Raje, 2019; van den Berg et al., 2019).

In order to implement such a complex concept, physiologic functions need to be implemented, such as breathing or muscle contraptions (Zheng et al., 2016). One of the more complex experiments realized is maintaining a certain glucose level by the use of liver and pancreatic cells (Wikswo et al., 2013; De Miollis et al., 2019).

The main drawback of the personalization of the OoC is that due to its complexity, some processes cannot be implemented yet and due to the specifics, it can be realized only with specific equipment.

## 5 Conclusion

The concept of OoC has been around for a long time and they have led to a lot of new discoveries in the medical field. The applications range from disease studying to drug testing considering the complex environmental interaction among different cell types. In the present times single-organ-on-a-chip models have already been created for almost all organs, with some studies on multiple OoC devices interconnected (Goldstein et al., 2021). One of the next development steps is the integration of sensors into the chips, which makes monitoring key physiological parameters easier (Clarke et al., 2021; Rothbauer et al., 2021). It is an important field which with further development can lead to new and revolutionary discoveries. The human-body-on-a-chip can be considered one of them. In most cases the medicine used to cure a certain type of disease may help in the recovery of a certain organ, but there are side effects which may occur in other organs. Having a model of the human body which consists of a system of OoC connected could put an end to animal testing and would mean a speed-up in the pharmaceutical industry. New biomaterials and techniques which may allow some of the harder and more complex physiological functions to be modeled with ease. For the fabrication of the OoC 3D printing, soft lithography, hot embossing and injection molding are all commonly used and each has some advantage over the other (Tajeddin and Mustafaoglu, 2021). Recently the concept of bioprinting has been used by many researchers which could lead to more cost-efficient fabrication of the OoC. Soft-lithography and photolithography, cost, time and design improvements are all more advantageous if bioprinting is used (Carvalho et al., 2021; Thakare et al., 2021). More importantly the different paths that are opened can reach to the automation of medical procedures and the elimination of mistreatment.

## Abbreviations

BoC, body-on-a-chip; PDMS, polydimethylsiloxane; ToC, tumor-on-a-chip; ECM, extracellular matrix; OoC, organ-on-a-chip.
